# 100 YEARS OF VITAMIN D: Dose–response for change in 25-hydroxyvitamin D after UV exposure: outcome of a systematic review

**DOI:** 10.1530/EC-21-0308

**Published:** 2021-09-13

**Authors:** Ann R Webb, Rehab Alghamdi, Richard Kift, Lesley E Rhodes

**Affiliations:** 1 Department of Earth and Environmental Sciences, University of Manchester, Manchester, UK; 2 Department of Clinical Nutrition, Faculty of Applied Medical Sciences, King Abdulaziz University, Jeddah, Saudi Arabia; 3 Centre for Dermatology Research, School of Biological Sciences, The University of Manchester and Salford Royal NHS Foundation Trust, Manchester, UK

**Keywords:** ultraviolet radiation, vitamin D, dose–response, bone, solar radiation, systematic review, 25-hydroxyvitamin D, skin, humans, in vivo studies, nutritional guidance

## Abstract

A systematic review of publications addressing change in vitamin D status (25-hydroxyvitamin D (25OHD)) after exposure to UV radiation identified 2001 independent peer-reviewed publications. Of these, 21 used artificial sources of UV radiation, met all inclusion criteria and were quality assured; 13 publications used solar radiation and met sufficient inclusion criteria to be retained as supporting evidence; 1 further included publication used both solar and artificial sources. The review consistently identified that low dose, sub-erythemal doses are more effective for vitamin D synthesis than doses close to a minimum erythema dose; increasing skin area exposed increases the amount of vitamin D synthesised although not necessarily in a linear manner; constant dosing leads to a dose-dependent plateau in 25OHD, and dose–response is greatest at the start of a dosing regime; there is a large interpersonal variation in response to UV exposure. Fourteen of the studies using artificial sources of radiation were used to determine a dose–response relationship for change in 25OHD on whole-body exposure to repeated sub-erythemal doses of UV radiation, taking the form Δ25OHD (nmol/L) = A ln(standard vitamin D dose) + B. This helps quantify our understanding of UV as a source of vitamin D and enables exposure regimes for safe synthesis of vitamin D to be assessed. Specific studies of people with pigmented skin (Fitzpatrick skin types 5 and 6) were rare, and this dose–response relationship is only applicable to white-skinned individuals as skin type is a determinant of response to UV radiation. Findings provide information for vitamin D guidance updates.

## Introduction

It is established that vitamin D is an essential part of maintaining a healthy musculoskeletal system, and it is hypothesised to play a role in a range of other diseases including support for the immune system (1).

There are two routes to acquiring vitamin D, by ingestion either through the diet or as supplement and through cutaneous synthesis initiated by the exposure of skin to UV radiation, specifically its UVB component. Modern diets do not, in general, provide for the body’s vitamin D needs, leading to public health decisions to fortify certain foodstuffs (2) or to recommend vitamin D supplements for some or all of the population (3, 4, 5) in light of evidence that large proportions of the population have low vitamin D status some, or all, of the time (6, 7). Given the often stated assessment that 90% of the body’s vitamin D supply is synthesised within the skin, low vitamin D status, determined by circulating 25-hydroxyvitamin D (25OHD) level, also implies a significant lack of exposure to the UV in sunlight. However, encouraging more sun exposure contradicts long-running health campaigns, at least in white-skinned populations, to restrict UV exposure and therefore sunburn, which acts as proxy for skin cancer risk. While ‘little and often’ sun exposure for the benefits of vitamin D status can avoid the majority of skin cancer risks, providing such a public health message is complex, especially as ‘little and often’ requires a personal prescription dependent on an individual’s characteristics and location. More heavily pigmented people (brown- or black-skinned) retain their melanin protection against UVB even when ambient sunlight levels are low. Thus, they require more sun exposure than their white-skinned neighbours to serve their vitamin D needs, yet they often retain cultural behaviours of sun avoidance. Despite these complexities, to assess needs or modifications to the ingested route for vitamin D, supply through cutaneous synthesis must be understood and quantified.

### Question to be answered

This systematic review aims to quantify the effects of UVB exposure on vitamin D status. An ideal outcome would be a dose–response curve for UVB dose and change in vitamin D status response, but there are many confounders and caveats to such a dose–response curve, as detailed below.

### Complexities of the vitamin D pathway: from sunlight to status

**Figure 1:**
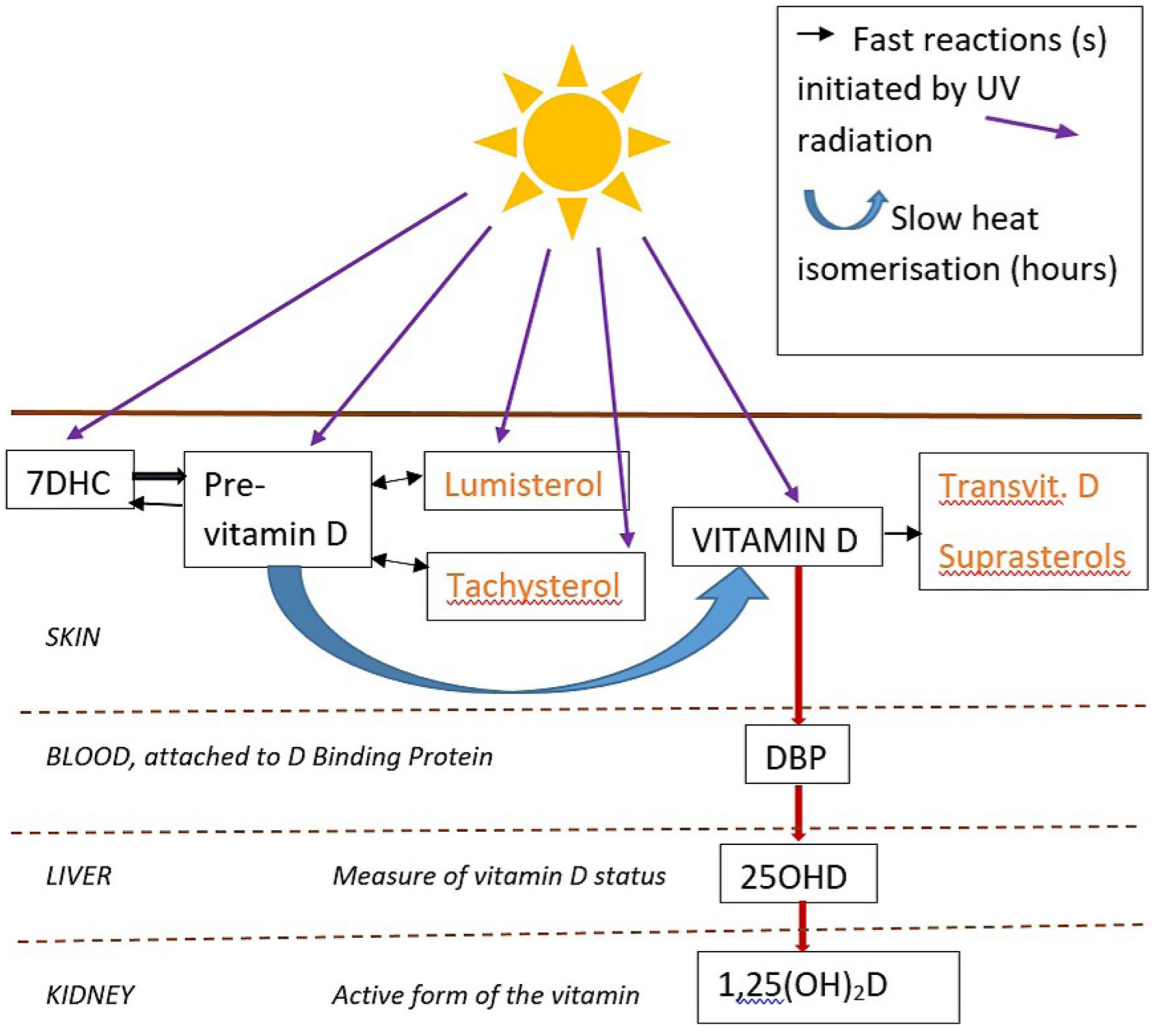
The cutaneous synthesis of vitamin D and path to its measured and active forms. UV radiation (specifically UVB), naturally present in sunlight, falling on unprotected skin, initiates vitamin D synthesis when it causes 7-dehydrocholesterol to photoisomerise to previtamin D. This is a rapid reaction. Thereafter, a slow heat isomerisation taking several hours results in the change from previtamin D to vitamin D. The vitamin D binds to a vitamin D binding protein and enters the circulation, from whence it is hydroxylated in the liver to form 25-hydroxyvitamin D, the measure of which is used to define vitamin D status. There are therefore several steps between the delivery of a dose of UVB to the skin, and the measurement of the response quantified as a change in the circulating 25OHD. The basic pathway is represented by black text in boxes. Photoproducts in orange are biologically inert and limit the supply of vitamin D despite prolonged exposure to UV radiation. 7DHC, 7-dehydrocholesterol; DBP, D binding protein; 25OHD, 25hydroxyvitamin D; 1,25(OH)_2_D, 1,25 dihydroxyvitamin D; Transvit., transvitamin.

Along the pipeline from UVB exposure of the skin to circulating 25OHD, several interruptions can occur. These are illustrated in Fig. 1 where the photoisomers (orange) of either previtamin D or vitamin D can, with prolonged exposure, accumulate at the expense of their parent isomer and reduce the end product of vitamin D (8, 9). The isomer mixture, especially around previtamin D, is also dependent on the spectrum of the irradiating source (10).

Vitamin D enters the circulation attached to vitamin D binding protein (DBP). DBP is produced in the liver and binds all circulating vitamin D metabolites, with greatest affinity for 25OHD (11), the hydroxylated form of the vitamin. This maintains a large circulating pool of 25OHD, reducing the impact of irregular supply of vitamin D (11). A further hydroxylation in the kidney to 1,25-dihydroxyvitamin D (1,25OH_2_D) is tightly controlled by the endocrine system and varies little with 25OHD status, until reaching clear deficiency.

In a controlled clinical environment, the change in 25OHD to a known UV dose is (usually) measured 24 h after UV delivery. Quantifying the response to solar UV received over a prolonged period in daily life is more challenging. A single measure of 25OHD represents a dynamic balance between supply of vitamin D from skin and gut, storage in adipose/other tissue, and use. Given the half-life of circulating 25OHD of several weeks, that single measure represents the multiple sources and sinks of vitamin D integrated over the previous weeks/months.

There are two major personal characteristics that can affect the skin’s ability to synthesise vitamin D upon UV exposure. First, skin type, or the amount of melanin in the epidermis, which is evolutionarily matched to solar intensity at the location of historical habitation. White-skinned immigrants at low latitudes have a greatly increased risk of sunburn and over time skin cancer; dark-skinned immigrants at high latitudes have a high risk of vitamin D deficiency, if relying on the sun as a source.

Secondly, age may influence vitamin D supply through the skin. Ability to synthesise vitamin D is thought to decline because the skin content of the precursor, 7-dehydrocholesterol (7DHC), reduces with age (12). However, it is unclear whether 7DHC or UV exposure is the limiting factor for the ambulatory older adult. While elderly care home residents were reported to show low vitamin D status (13, 14), they may also have very limited access to sunlight; in healthy older adults spending regular time outdoors, vitamin D deficiency can be avoided (15).

Finally, the Commission Internationale de l'Eclairage (CIE) action spectrum for the conversion of 7DHC to previtamin D (16), used here to quantify all sources of UV radiation in comparable units, is not universally accepted. It has been questioned (17, 18) and other action spectra suggested (19), but to date, no alternative has proven better supported by experimental outcome than the CIE action spectrum (20).

The review was commissioned to inform the updating of Food and Agriculture Organisation-WHO nutrient requirements.

## Methods

### Systematic review

The preferred reporting items for systematic reviews and meta-analyses guidelines were used to aid reporting.

### Search criteria and databases

The databases searched were Embase (including MEDLINE https://www.elsevier.com/solutions/embase-biomedical-research) and Cochrane Central (https://www.cochranelibrary.com/central/about-central). Searches were conducted on 19 October 2020, and eligible publications from the date of inception of the database were identified, without limitation on language.

Search terms for all databases required a term for vitamin D/25OHD plus a term for sunlight or UV radiation. For Embase and MEDLINE, an additional term indicating a human trial or study was required. Animal trials were excluded, as were conference abstracts. Full details of search terms are given in Supplementary data (see section on supplementary materials given at the end of this article).

**Figure 2:**
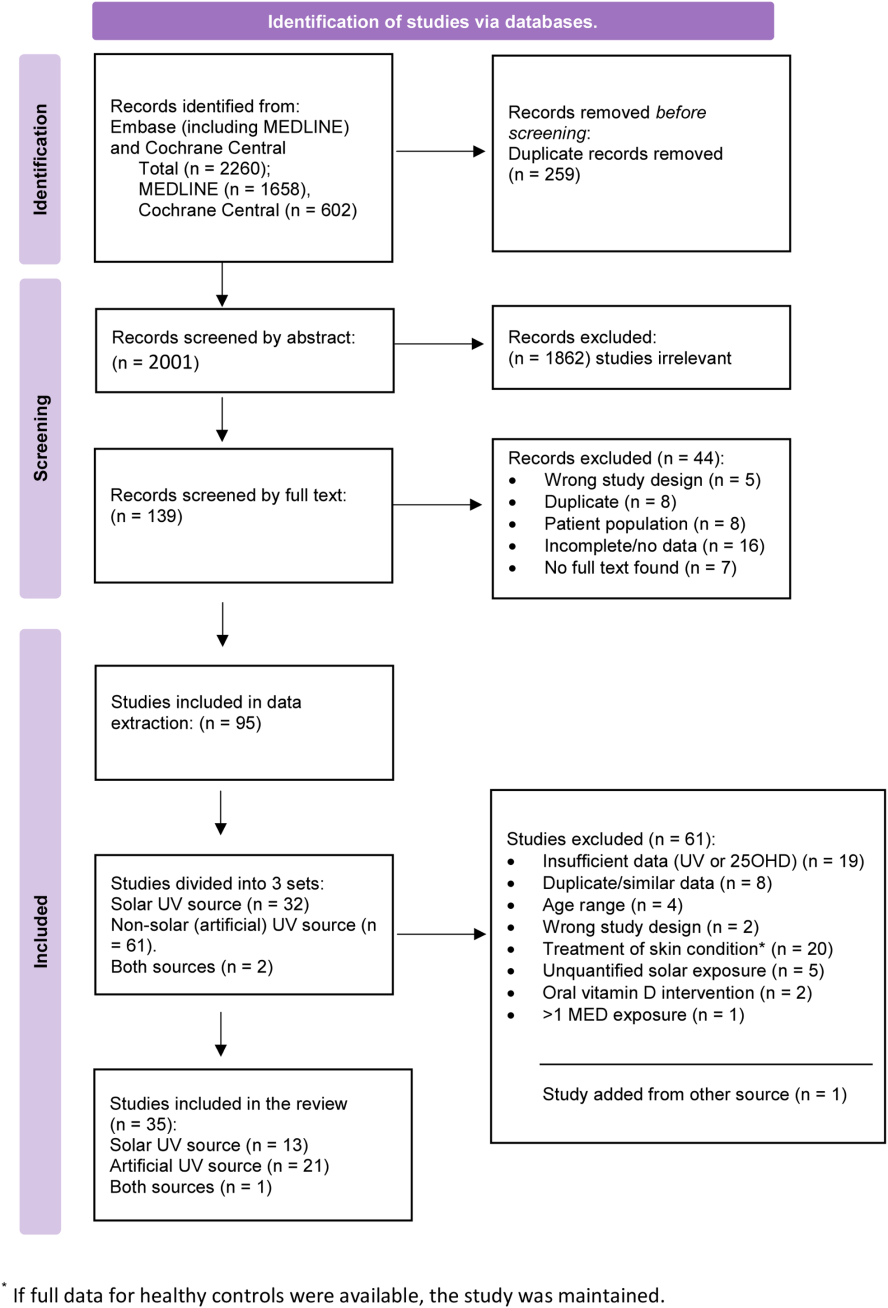
Flow diagram of record screening and data extraction.

The initial Embase and MEDLINE search returned 1658 eligible records while Cochrane Central returned 602 (Fig. 2). On combination, 259 duplicates were removed leaving 2001 unique records to screen. These were uploaded to the Covidence system (https://www.covidence.org/) through which the screening was managed.

### Screening criteria and screening results

Inclusion criteria were healthy children and adults (<65 years age) of any skin type; a quantified measure of UV exposure that was <1 minimal erythema dose (MED), that is, below the threshold dose for sunburn erythema, per dose; baseline and outcome 25OHD levels stated; intervention studies, including randomised control trails (RCTs), non-randomised control trials and non-controlled (before–after) interventions. Exclusion criteria were pregnancy or lactation; any illness that might impact vitamin D or calcium status or metabolism; observational, case–control, cross-sectional, ecological and animal studies; reviews. Observational studies involving sun exposure and meeting all other criteria were later added solely to contextualise the intervention study findings (mainly conducted with artificial sources of UV). Only peer-reviewed journal publications with full text available were included. (Full inclusion/exclusion criteria are in Supplementary data).

Initial screening of titles and abstracts (by ARW and KC) resulted in 139 publications, reduced to 95 at initial full-text screening prior to data extraction. The majority of exclusions at the full-text stage were trial abstracts whose resulting publications had either already been captured or are not yet available, the wrong study type (cross-sectional/observational), or patient volunteers. The process of selecting included studies is shown in Fig. 2.

### Data extraction criteria

Manuscripts were separated into three sets for data extraction (RA and RK) – 32 where solar radiation was the source of UV, 61 using non-solar sources of UV, hereafter referred to as artificial UV, and 2 studies that documented both sources. Any uncertainties were resolved on discussion with a further author.

The solar exposure papers were further reduced to 12 (21, 22, 23, 25, 26, 27, 28, 29, 30, 31, 32, 33), mainly by lack of sufficient information on UV exposure (e.g. ‘normal lifestyle’ with no quantification) and/or change in 25OHD associated with specific exposures. Most studies worked with adult volunteers, but there were two groups of adolescents, and a small amount of work with infants (age <1 year). Solar exposure is quantified in one of two ways: by dosimetry or description of time in the sun. Dosimetry does not provide any indication of skin area exposed or use of sun protection, so supplemental information is required to make the best use of the data. Time in the sun, either quantified via a sun exposure diary or through following a prescribed behaviour, also requires substantial additional detail to enable an accurate dose to be determined (location, time of day, weather, skin exposure and sunscreen use, with an indication of behaviour, e.g. sitting in full sun/under a tree). Both methods of quantifying exposure are subject to challenges of compliance. Despite the complexities of determining sun exposure, it is an individual’s relationship with sun exposure over the course of the year that will have a major impact on their vitamin D status at any point in time. Therefore, studies were selected for analysis where there was a clear attempt to quantify unprotected skin exposure, by intermittent dosimetry, exposure diary or prescribed time and hour of day in the sun. To this is added the appropriate data from the remaining record that used both solar and artificial sources of UV (24). In addition, we added a study not originally identified in our search but meeting the above criteria in a large sample, albeit that 25% of the participants were older than 65 years (34).

Artificial UV exposure manuscripts (including data from the record using both sources (24)) were reduced to 22 (20, 24, 35, 36, 37, 38, 39, 40, 41, 42, 43, 44, 45, 46, 47, 48, 49, 50, 51, 52, 53, 54) mainly due to lack of sufficient information on UV source deployed, the exposure regime and/or lack of before and after 25OHD measurements. All remaining studies represented work with adult volunteers, provided with a course of standardised, artificial UV exposures, but UV source and dosing regime varied significantly between studies. They were first stratified with respect to type of UV source used, that is, narrowband UVB (NB-UVB), broadband (BB) or simulated solar radiation (SSR), and within that by skin type of volunteer. The majority of studies used White Caucasian volunteers (skin types 1–4), two studies looked exclusively at skin type 5, and one at skin type 6. Several studies included volunteers across the full range of skin types.

The dosing regimes for artificial UV were varied and specified in different ways. Many of the trials used a standard phototherapy dosing regime, beginning with a low dose (skin type or MED dependent) and increasing this steadily with each dose to a defined maximum, or until adverse effect (e.g. erythema). In these cases, individual dose regimes were not provided, and at best the treatment was summarised by a mean or median cumulative dose over the treatment period. The most common exposure regimes were two or three irradiations per week for 8 or 12 weeks, with this level of variation existing within some studies without any further breakdown in reporting results. However, across all studies, irradiations ranged from a single dose to repeated dosing over 24 weeks.

Artificial UV studies did not record solar UV exposure, but the vast majority were conducted during the winter, when the effects of solar exposure are expected to be negligible. The main exception is the study by McKenzie *et al.* (20) which is discussed where appropriate. Dietary vitamin D was not routinely determined in most studies, though supplement use was usually either an exclusion factor, recorded, or in one case (39) the UV regime was in addition to prescribed supplements.

### Quality assessment of studies

The methodological quality of the 22 studies using artificial sources of UV was assessed by two researchers (RA and ARW), and scoring was further checked (LER), according to a 12-point (before–after studies) or 14-point scale (RCTs), to determine risk of bias as high, medium or low (https://www.nhlbi.nih.gov/health-topics/study-quality-assessment-tools).

The solar UV studies have been retained as supporting data rather than as inputs to the dose–response curve and were therefore not quality assessed in this manner.

### Combined dose–response

An aim of this systematic review was to explore a dose–response relationship between UV dose and change in circulating 25OHD (Δ25OHD). Given the very different spectra of the artificial UV sources used, and in turn their difference from sunlight, combining data from different sources is only valid if all UV doses are converted to vitamin D effective (VDE) UV, that is the spectral UV of the source weighted by the vitamin D action spectrum (16). However, the vast majority of studies provided a UV dose in units of SED, where 1 SED is 100 Jm^−^^2^ of erythema effective (EE) UV (55). If the spectrum of the irradiating source is known, then the ratio of VDE:EE UV (VDE:EE) can be calculated and applied to the doses delivered. The vast majority of publications did not provide either full spectral detail of the specific lamps used or information on the VDE dose. Therefore, we have calculated the VDE:EE ratios for typical lamp models, using measurements of phototherapy units in Manchester. Where other authors have also made this calculation, the data are shown in Table 1 to indicate the degree of consistency in the ratio for the lamp type. The mean of all available ratios for a lamp type was applied to all doses specified in SED for that lamp type, to provide VDE doses, except in cases where the original authors supplied conversion factors of their own

**Table 1:** The ratio of vitamin D effective UV to erythema effective UV (VDE:EE) for a range of standard UV sources.

Source	VDE:EE (Manchester)	VDE:EE (other authors)	VDE:EE (mean)
TL-01	2.15	2.3 (24); 2.27 (20)	2.24
TL-12	1.29	1.3 (32); 1.16 (20)	1.25
UV6	1.48	1.62 (59)	1.55
Arimed B	1.83		1.83
Wolff Life Sun	1.85		1.85
Sun	1.89 (summer)	2.01 (NZ summer (20)); 1.9–1.99 (summer (32))	
		1.22 (NZ winter (20))	

The solar spectrum is not constant; it changes with solar elevation and to a lesser extent with column ozone amount across the UVB part of the spectrum. The VDE:EE ratio therefore changes with solar elevation and ranges from about 2 at low latitudes/mid-summer/noon to around 1 or below at high latitudes/mid-winter/sunrise or sunset. Study (20) illustrates this with the VDE:EE ratios of noontime sunlight in New Zealand, these being 1.22 in winter and 2.01 in summer. In Manchester, UK, the equivalent figure is 1.89 (midsummer noon). Given that low elevation sunlight is ineffectual for vitamin D synthesis and also represents a small part of most summer day doses, a representative VDE:EE ratio of 1.89 was used for Manchester data and locations at equivalent latitudes, while 2.0 was used for locations closer to the equator.

Having converted all data from artificial UV sources to VDE doses, data from all studies with 'whole-body' exposure (i.e. just eyes and genitalia covered, ~90% skin surface area exposed) at sub-erythemal doses were combined to provide a single dose–response relationship. (56) has suggested that a dose ~1 MED marks the point where production and loss of vitamin D are balanced, suggesting both an exponential response to UV and a limit to the dose–response. Furthermore, UV doses that produce erythema are not to be encouraged as they clearly induce skin damage. The VDE doses are reported in standard vitamin D dose (SDD), which by analogy with SED is taken as 100 Jm^−^^2^ VDE radiation (57).

### Statistical details

Data have been extracted with the statistical detail provided by the original authors. Values in tables are averages: in most cases, this is the mean, but some studies provided a median. Due to the non-linearity of the 25OHD response to UV exposure of the skin, we used a linear log plot for the dose–response, in conjunction with a logarithmic line fit to minimise residuals and display a linear relationship between the change in 25OHD and SDD (58).

## Results

**Figure 3:**
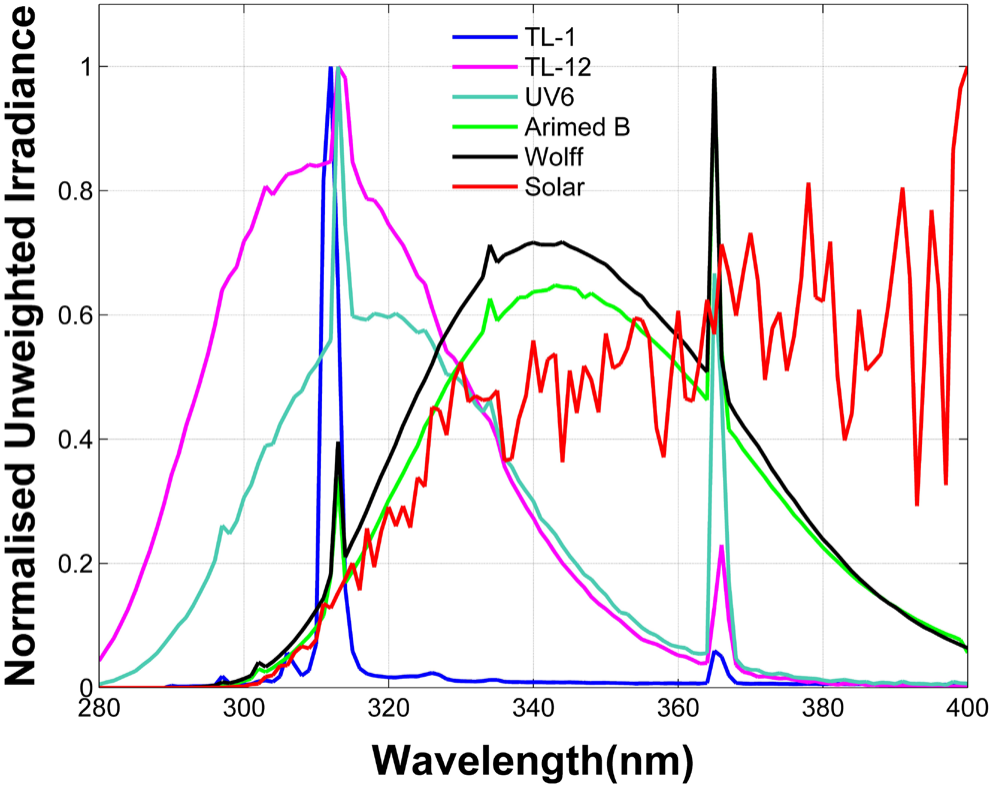
Spectra of commonly employed artificial UV sources and a summer solar UV spectrum measured in Manchester, UK. Spectra have been normalised at peak wavelength for each source. Source data for the artificial sources of UV courtesy of Dr Donald Allan, University of Manchester.

Of the original 2001 searched publications, a total of 22 studies with artificial sources of UV radiation and 14 supporting studies with solar radiation were retained after all screening and data extraction requirements had been met (Fig. 2). The 22 artificial source works consisted of 12 RCTs and 10 interventional before–after studies. With the exception of one single dose, before–after study, all solar studies were observational, and either monitored solar exposure or prescribed a time of day and duration for solar exposure. Extracted data are shown in Tables 2, 3, 4 and 5 which provide numbers and demographics of volunteers in subsections according to UV source. Data are from studies with healthy volunteers as the main focus or as healthy controls in comparison with patients. Figure 3 shows example spectra of the sources.

**Table 2:** NB-UVB treatment studies using TL-01 lamps on healthy subjects. QA is quality assessment score giving low, medium or high risk of bias. *N* is the number completing the study, if detailed and different from those recruited. Data in normal type are taken directly from the original publication, and where averaged is the mean or median as provided by the original publication. Data in italics have been inferred or calculated by current study authors. Where the original 25OHD was given in ng/mL, this has been converted to nmol/L (×2.5) without further noting the change.

Study (QA score)	*N*	Skin type	Total dose SED	Total dose SDD	Change in 25OHD nmol/L	nmol/L/SED	nmol/L/SDD
Karppinen *et al*. (35) (low)	16	2–4	*25* *19*	*57.5* *42.6*	11.7 26.2^a^	*0.47* *1.38*	*0.20* *0.61*
Bogh *et al*. (36) (medium)	16	2–6	*52.9*	*118.5*	55.8	*1.05*	*0.47*
Bosman *et al*. (37) (low)	12F −S^b^ 9F +S	1–3	*3.5*	*7.84*	10.1 3.8	*2.88* *1.08*	*1.29* *0.48*
Ponda *et al*. (38) (medium)	58	1–6	*38.7*	*86.7*	35^c^	*0.90*	*0.40*
Ala-Houhala *et al*. (39) (low)	15 +S2^b^	2–4	25.7	*57.6*	17	*0.66*	*0.29*
Ala-Houhala *et al*. (40) (low)	33	2–4	48.4	*108.4*	41	*0.84*	*0.38*
Mckenzie *et al*. (20)^d^ (low)	58	1-3,4,5,6	268	608	*48.9 Raw* 62.8 Adj.	*0.18e* *0.23e*	*0.08e* *0.10e*

^a^Maximum change in 25OHD observed after 20 weeks, after which values declined with dosing for a further 6 weeks; ^b^−S, no supplement, +S, course of supplements (1000 IU+/day) prior to dosing, +S2 800 IU/day for average 3.4 months prior to dosing; ^c^after 2 months consistent treatment, maintenance phase not included; ^d^study (69) reports on the same participants in the same study, but study (20) provides more detail on the radiation sources; ^e^raw data calculated (by the current authors) from Table 4 of the original study (20). Adjusted data (adjusted by the original study authors) are taken from Table 5 of study (20) and represent a modelled adjustment for exposure to solar radiation. The original study (20) quotes the 'adjusted sensitivity' as 0.4 nmol/L/SED and 0.2 nmol/L/SDD, these being the means of the individual participant sensitivities, and not calculated, as here, from mean data in Tables 4 or 5 of the original publication (R Mckenzie, personal communication).

**Table 3:** Broadband treatment studies using TL-12 and UV6 sources. QA is quality assessment score giving low, medium or high risk of bias. *N* is the number completing the study, if detailed and different from those recruited. S.A. is skin surface area exposed, and = ‘whole body’ (eyes and genitalia covered) unless otherwise specified. Data in normal type are taken directly from the original publication, and where averaged is the mean or median as provided by the original publication. Data in italics have been inferred or calculated by current study authors. Where the original 25OHD was given in ng/mL, this has been converted to nmol/L (×2.5) without further noting the change.

Study	*N*	Skin type, S.A.	Total dose SED	Total dose SDD	Change in 25OHD nmol/L	nmol/L/SED	nmol/L/SDD
Source (QA)
Bogh *et al*. (41) TL12 (low)	55	1–4 24%	1.5 3.0 6.0 12.0	1.95 3.90 7.80 15.60	14.2 19.9 18.6 24.8	*9.47* *6.63* *3.1* *2.07a*	*7.28* *5.1* *2.38* *1.59*
Osmancevic *et al*. (42) TL-12 (high)	2 1	6	*481* *436*	*601* *545*	30 6	*0.06* *0.014*	*0.05* *0.01*
Bogh *et al*. (43) TL-12 (low)	92	1–4 6%,12% or 24%	3.0 6.0 12.0	3.90 7.80 15.60	1.9–19.9 13.5–19.7 22.7–25.0 (6–24%)	*6.63* *3.28* *2.08a (24%)*	*5.30* *2.62* *1.60 (24%)*
Mckenzie *et al*. (20) TL-12 (low)	61	1–3,4,5,6	17.1	19.8	*19.5 Raw* 31.7 Adj.	*1.14b* *1.85b*	*0.98b* *1.60b*
Sallander *et al*. (44) UV6 (medium)	23 23A	1–5	*2.62* *2.7*	*4.25* *4.38*	*11.6* *13.6*	*4.43* *5.03*	*2.73* *3.10*
Datta *et al*. (24) UV6 (low)	22	2–4 80%	*19.24*	*29.8*	49	*2.49*	*1.60*
Yesudian *et al*. (45) UV6 (medium)	8M	5	21	*32.5*	22.9	*1.09*	*1.69*
Bogh *et al*. (46) UV6 (high)	15^c^ 14 12	1–4	17^c^ 9 5	*26.35* *13.95* *7.75*	12.6 −4.7 −8.6	*0.74* *Insuff* *Insuff*	*0.48*

M, male participants; A, UVB lamps supplemented with UVA lamps not considered to initiate vitamin D synthesis; ^a^same participants and doses reported in two publications; ^b^raw data calculated (by the current authors) from Table 4 of the original study (20). Adjusted data (adjusted by the original study authors) are taken from Table 5 of study (20) and represent a modelled adjustment for exposure to solar radiation. The original study (20) quotes the 'adjusted sensitivity' as 2.2 nmol/L/ and 1.9 nmol/L/SDD, these being the means of the individual participant sensitivities, and not calculated, as here, from mean data in Tables 4 or 5 of the original publication (R McKenzie, personal communication); ^c^Number of participants in three groups receiving three different exposure regimes (1 SED once per week, once every 2 weeks, once every 4 weeks for 16 weeks); Insuff, dose insufficient to increase 25OHD.

**Table 4:** Studies using solar-simulated radiation (SSR), from a range of similar sources. QA is quality assessment score giving low, medium or high risk of bias. *N* is the number completing the study, if detailed and different from those recruited. S.A. is skin surface area exposed, and = ‘whole body’ (eyes and genitalia covered) unless otherwise specified. Data in normal type are taken directly from the original publication, and where averaged is the mean or median as provided by the original publication. Data in italics have been inferred or calculated by current study authors. Where the original 25OHD was given in ng/mL, this has been converted to nmol/L (x2.5) without further noting the change. Dose was scaled to skin type – that given is for the majority, skin types 3 and 4.

Study	*N*	Skin type S.A.	Total dose SED	Total dose SDD	Change in 25OHD nmol/L	nmol/L/SED	nmol/L/SDD
Source (QA)
Rhodes *et al*. (47) Arimed B+ Cleo Natural (low)	109	1–4 35%	23.4	*42.8*	26.0	*1.11*	*0.61*
Langdahl *et al*. (48) Cleo Swift (low)	11	2,3	*4.55*	*8.32*	4.5	*0.99*	*0.54*
Farrar *et al*. (49) Arimed B (low)	51	5 35%	*11.7* *23.4* *35.1* *46.8* *58.5* *70.2*	*21.4* *42.8* *64.2* *85.6* *107* *128.5*	9.0 11.5 16.8 16.8 31.5 23.8	*0.76* *0.48* *0.48* *0.36* *0.54* *0.34*	*0.42* *0.37* *0.26* *0.20* *0.30* *0.19*
Felton *et al*. (54) Arimed (low)	10 6	2 5 35%	23.4 23.4	*42.8* *42.8*	17.8 8.3	*0.76* *0.35*	*0.42* *0.19*
Biersack *et al*. (50) Arimed (low)	2F 18F	1 2,3	*3.5* *4.375*	*6.4* *8.0*	13.9	*3.18*	*1.74*
Lagunova *et al*. (51) Wolff (high)	11 11^a^	1–4	23.8 23.8	*44.0* *44.0*	19.8 5.1^a^	*0.83* *0.21a*	*0.45* *0.11a*
Carbone *et al*. (52) Wolff (medium)	15^b^ 10^b^	2–5	30.24	*55.9*	*85* *30*	*2.81* *0.99*	*1.52* *0.54*
Porojnicu *et al*. (53) Wolff (low)	10	1,2	*18.7 SED* *12.1SEDc*	*34.2* *23.2c*	*26*	*0.76* *1.17c*	*0.41* *0.63c*

^a^Cross-over study, second arm had received a period of supplementation before UV dosing; ^b^participants divided into those with mean starting 25OHD of 50 nmol/L (top 15) and mean starting 25OHD of 122 nmol/L (bottom 10); ^ c^25OHD measured weekly, this is the dose at which 25OHD first plateaued.

**Table 5:** Solar (sun exposure) studies, using either seasonal dosimeter data, or specified time in the sun. *N* is the number completing the study, if detailed and different from those recruited. Data in normal type are taken directly from the original publication, and where averaged is the mean or median as provided by the original publication. Data in italics have been inferred or calculated by current study authors. Where the original 25OHD was given in ng/mL, this has been converted to nmol/L (x2.5) without further noting the change.

Study	*N* Skin type/ethnicity	UV measure	Location	Total dose SED	25OHD (nmol/L)	Summer–Winter (nmol/L)	Δ25OHD/exposure (nmol/L/SDD/time)
	Age^a^		Duration				
Darling *et al*. (21)	90F 1–4	PS, 1 wk/season	Surrey, UK (51.3N) Year-round	6.39 S 0.64 W	72.1 S 44.5 W	*27.6*	*2.29/summer week*
Rhodes *et al*.^b^ (22)	109 1–4	PS, 1 wk/season	Manchester, UK (53.5N) Year-round	3.49 S 0.12 W	68.0 S 45.5 W	*22.5*	*3.41/summer week*
Farrar *et al*. (23)	115S 90W 1–4 13–15 yr.	PS, 1 wk/season	Manchester, UK (53.5N) Year-round	3.1 S 0.1 W	60.3 S 38.8 W	*21.5*	*3.67/summer week*
Darling *et al*. (21)	35F 5	PS, 1 wk/season	Surrey, UK (51.3N) Year-round	2.00 S 0.29 W	26.2 S 19.7 W	*6.5*	*1.72/summer week*
Datta *et al*. (24)	19 2–4	Sunsaver, 1 wk total	Egypt (27.2N) 1 wk, after UV6 trial	*66 kJm−2 (with s = 22)c*		*−3*	*Insufficient*
Rueter *et al*. (25)	48 80% Mothers White (0–6 mos.)	Viospor 0–3 mo. (F+H+A)	Perth, AUS (31.9S) 3 mos.	815 Jm^−^^2 c^	76.0 maternal 59.2 (infant)	*−16.8*	*Insufficient*
Pereira *et al*. (33)	10 n 64 s 1–5		Rio de Jan. Brazil (22.9S) 24 h.	*2SED* (20 mJ/cm^2^)		10.3 13.8	*2.6/SDD* *3.5/SDD*
Scragg *et al*. (34)	512 1–4 18–85 yr.	Personal electronic dosimeter 4 wk	Auckland (37S) and Dunedin (46S)	*2.04*	48.2 start 47.4 end	*−0.8*	*Insufficient*
		**Time in the sun**				**End start**, nmol/L	
Meena *et al*. (26)	100 3,4 2–6 mo.	17 min 6% skin area	Delhi, India (28.7N) 6 mos.		15.8 Maternal 23.0 (Infant)	7.2	
Patwardhan *et al*. (27)	32M 5	20 min 11-3	Pune, India (18.5N) 6 mos.		35.6 48.3	12.7	
Ho *et al*. (28)	18 Chinese 1–8 mo.	~2 h/d (F+H)	Beijing, China (39.9N) Sep–Oct		70 100	30	
Watcharanon *et al*. (29)	26F 4	30 min @3 pm	Khon-Kaen, Thailand (16.4N) Jun–Sept		80.8 74.2	−6.6	*Insufficient*
Dawodu *et al*. (30)	8F Arab	15 min 2/wk	Al Aen, UAE, (24.1N) 4 wk		17.6 23.0	5.4	
Wicherts *et al*. (31)	47 4–6	Diary 120 min	Netherlands (52.1N) Mar–Sept		23.3 *~33 (J)* *~26 (S)*	*9.7 (J)* *2.7 (S)*	
Joh *et al*. (32)	49 1–6 (mainly 3,4)	Diary >20 min	Seoul, Korea (37.6N) 8 wk (rolling)		24.0 *34.5(4)* *35(8)*	12.7 for >30 m	

^a^Age if not adult (18–65 years), study (34) had 25% participants aged 65–85 years, data inseparable from younger ages; F, female; M, male; S, summer; W, winter; PS, polysulphone dosimeter badge; *n* , unprotected; s, self-administered sunscreen; (F+H+A), face and hands and arms exposed; (J), outcome in June; (S), outcome in September; (4), outcome after 4 weeks; (8), outcome after 8 weeks; ^b^Study (22) uses data from a previous study ((68) White Caucasians) and compares them to a new cohort of photosensitive patients, though only the healthy cohort are described here. Similarly, study (21) compares Caucasian and South Asian postmenopausal women; ^c^Dose assumed in erythema effective units but not explicitly stated as such.

Quality assurance of the 22 studies using artificial sources of UV indicated that 2 were at high risk of bias, 5 had medium risk and the remaining 15 were at low risk of bias (Tables 2, 3 and 4).

### Narrowband-UVB studies

The NB-UVB studies all used phototherapy units, most commonly defined as ‘narrowband, 311 nm’ and identified as TL-01 lamps or equivalent. These are variously described as having an output covering the wavelength range 310–315 nm, with peak 311/312 nm, and 85% output between and 311–313 nm. A typical output spectrum is shown in Fig. 3.

Analysis of NB-UVB treatment was limited to seven studies on healthy adults or healthy controls undertaking treatment alongside patients (Table 2).

With the exception of study (35), all studies gave UV doses defined by skin type/MED and that increased steadily (according to different protocols) with time, stopping or pausing the increase for participant if there were adverse reactions. No study gave information about individual doses – at best there was an indication of the mean total dose provided. Where this was not given (37, 38), a crude estimate has been calculated by current authors based on information given in the paper. All dosing was to ‘whole body’, with eyes and genitalia protected.

The studies underscore a number of points about the response of 25OHD to vitamin D supply, either individually or in concert. Between studies lasting several weeks, those with the least response are studies (35) and (39) where starting 25OHD was already >70 nmol/L, while the greatest response was seen in study (36) where participants started with vitamin D deficiency (<20 nmol/L). High starting 25OHD was achieved by supplementing participants for at least 3 months before the study (39), or beginning at end summer when 25OHD is at a peak (35). The aim of study (35) was to determine whether summer 25OHD levels could be maintained throughout winter with a fortnightly dose of 2SED NB-UVB (for white-skinned people). This proved to be the case, with the control group observing −11.1 nmol/L change over the 6-month period. There is good agreement in response to this NB-UVB between studies (24) and (27), especially if the results at maximum 25OHD are taken from study (35): it has been shown previously (21, 22, 59) that with consistent dosing 25OHD levels plateau after several weeks. This is underscored by the two studies with the greatest duration and the smallest nmol/L/SDD at the final time point (20, 35).

The outlier in this group of studies is study (37), taking place over a single week. It indicates much greater response to NB-UVB than the other studies but does conform with the expectation that the greatest response to increased vitamin D supply is observed early in the period of increased supply. This study is also the one that internally confirms the impact of starting 25OHD status. Finally, the estimation of total dose should be treated with caution as it was not provided within the reference.

### Broadband UVB (TL-12, UV6) studies

These two common BB phototherapy sources are not identical, though they cover the same waveband range. They are more similar to each other than to either NB-UVB or sunlight – real or simulated (Fig. 3). They have a greater proportion of UVB radiation in their output than other BB sources, particularly at the short-wavelength end of the range. As Table 1 shows, the VDE:EE ratio is different for the two sources, and so the VDE dose is required even when comparing Δ25OHD values from these two sources. Details are shown in Table 3.

The studies used a wide variety of dose regimes, skin surface area exposed, skin type and duration, making them difficult to compare directly. Studies (41, 43) and (46) were exploring the effects of different dosing regimens, with studies (41, 43) reporting on some of the same participants; therefore, close agreement between these studies is not surprising. Comparisons of dosing regimes were made between small groups of different people. They show that small doses of UV on a regular basis (four doses, each 2–3 days apart) are more efficient for vitamin D synthesis than larger doses – increasing dose by a factor of 8 less than doubled the vitamin D response (41). Increasing skin area exposed (from 6% to 24%) increased the overall response in 25OHD, but, at least for the small groups concerned, this was most obvious at low doses of UV, while the dose–response was most clear at small skin areas.

Where changes in 25OHD are negative, the term ‘insufficient’ has been used for the response – that is, the dose was insufficient to cause a measurable positive change in 25OHD. Anything produced was less than the body’s use of available 25OHD. This is a similar situation to the ‘vitamin D winter’, that is, when 25OHD declines through the winter months because of low elevation sun, short days and little skin area exposure. It is not impossible to synthesise vitamin D in skin at these times, but biologically relevant amounts are unlikely and it is either impractical or impossible (depending on location) to gain enough to maintain summer vitamin D status throughout the winter.

Study (46) explored the winter-time dose regime that would be necessary to maintain summer vitamin D status throughout the winter. A single whole-body irradiation of 1 SED once per week resulted in a modest increase in circulating 25OHD for White Caucasian participants over a 4-month period, while the same dose given every 2 or 4 weeks did not maintain starting levels of 25OHD.

Other dose regimes lasting several months were given in studies (20) and (24), at 12 and 9 weeks, respectively. The average total dose (skin type-matched dosing across all skin types) for study (20) was very similar to that of study (46), but provided over 12 rather than 16 weeks, and with a regime of increasing rather than fixed doses, resulting in a greater overall efficiency of 25OHD production. Study (24) explored interpersonal differences in response to UVB exposure, which were considerable, for example, the mean change in 25OHD was 48 nmol/L, but the range across 22 participants of similar skin type, given the same increasing dose regime, was 3–139 nmol/L. The average response was similar to that of study (20) and for a comparable total dose. Personal responses supported a non-linear, albeit personal, dose–response (i.e. plateauing response at constant dose). 25OHD baseline level did not influence the slope significantly; this was expected as baselines were relatively high.

The majority of studies were conducted with White participants (skin type 1–4), though study (20) had participants classed as European, Maori and Pacific, covering the full range of skin types (1–6) with dose matched to skin type for the three groups. Only studies (45) and (42) studied solely the higher skin types, 5 and 6, respectively. Study (45) gave three whole-body irradiations of 7 SED to South Asian males on consecutive days and elicited a rise in 25OHD. The efficiency (nmol/L/SDD) is at first glance similar to that for some White Caucasian results, for example, studies (24, 41). However, the White Caucasian skin type results were achieved at lower dose on 24% skin area, or over 9 weeks respectively, rather than 3 days full-body exposure (45). Study (42) was the only one to study and analyse skin type 6 independently, but only three participants completed the study and those were split between two different skin areas (upper body or hands and face); the study is on the borderline of acceptability for this review (*N* ≥ 2) and also classified as high risk of bias. The rise in 25OHD over 12 weeks was significant and greater for two participants with the greater skin area exposed. The efficiency of 25OHD change per SDD was the lowest of all the studies – again as might be expected for a highly pigmented skin.

### Solar-simulated radiation (Arimed B/Cleo Natural/Wolff Life Sun) studies

These lamps, often identified as providing SSR, are closer to the solar spectrum than the previous lamp classifications, in particular the shape of the short UVB wavelength part of the spectrum that is most effective for erythema and vitamin D synthesis (see Fig. 3). The UVA portion of the spectrum is less like sunlight, the lamps containing a greater proportion of shorter-wavelength UVA (UVA2) radiation.

Extracted data from studies that used SSR cabinets are given in Table 4. Publications (47) and (49) are work by the same group, using the same protocols of three fixed doses per week for 6 weeks. Study (49) was a dose–response study in skin type 5 adults, for comparison with the White Caucasian study (47). It showed that to reach the same absolute change in 25OHD, skin type 5 individuals need 2.5–3 times larger UV doses as white-skinned individuals. All groups, at all doses and skin types, showed a plateauing effect, with the rate of change of 25OHD decreasing with time. The efficiency is calculated after the final irradiation and as expected is greater for white-skinned than skin type 5 individuals. In the dose–response study, the greatest efficiency was seen at the lowest dose, albeit this still resulted in the smallest absolute change. This concurs with studies (41) and (43) (Table 3) where the smallest, oft-repeated doses were most efficient at raising 25OHD.

Study (50) gave three doses on 3 consecutive days, which is similar to the first week of study (47) (three doses over 5 days), and with a similar total dose for 1 week. However, study (50) used whole-body radiation (~90% skin area) vs 35% for study (47). Given that the increase in 25OHD was greatest in the first weeks of study (47), these sets of results are reasonably consistent.

Study (48) provides a similar dose to study (50), both as whole-body irradiations. However, study (48) is provided in one dose, and at a level that must be close to an MED for the participants, leading to the lower efficiency observed with other radiation sources when the dose is (too) high (41, 43, 60).

Study (51) included some participants taking vitamin D supplements. It was a cross-over study with one group receiving whole-body irradiation twice per week for 5 weeks and the other group 2000 IU/day. Then the groups were swapped, meaning that the second irradiation group began with a much higher 25OHD status than the first group and experienced a more modest Δ25OHD as a result, supporting earlier observations. This publication deemed that whole-body irradiation twice per week (an initially increasing, but sub-erythemal, dose) was equivalent to 2000 IU/day in raising and then maintaining vitamin D status. The influence of starting 25OHD on response to UV radiation is again illustrated by study (52) when there is a clear difference in response between those starting with 25OHD averaging 50 nmol/L, compared to 122 nmol/L.

Finally, study (53) measured 25OHD weekly, both during the period of UV dosing and for 8 weeks afterwards. Table 3 shows Δ25OHD/SDD both for the peak 25OHD and after all doses. As in previous studies, the 25OHD increased and then plateaued as dosing continued, so efficiency of UV dosing is greatest if calculated when maximum 25OHD is first reached. Once dosing was complete, this trial split the participants into two groups, one receiving 200 IU/day vitamin D and the other with no supplement. Both groups saw a decline in status, almost back to baseline, after 8 weeks, with no statistical difference between the (small, *n* = 5) groups, although the supplemented group had marginally higher 25OHD levels than the unsupplemented group. The conclusion was that 200 IU/day is insufficient supplement to maintain post-irradiation vitamin D status.

### Solar exposure studies

Studies using the sun as UV source and including some quantified measure of exposure, plus changing vitamin D status related to the exposure, have been included in the analysis for comparison but are insufficiently defined to be included in a dose–response and have not been quality assessed. The works fall into two groups, those using dosimetry to quantify UV exposure and those describing it by time in the sun at location. For most of the latter, there is insufficient information to enable UV in SED, or SDD, to be quantified, and the results must be discussed in terms of what local sun exposure can do for the vitamin D status of local people (with skin type typical of region). Data are provided in Table 5. Solar studies are also less well defined in terms of skin area exposed, although it is clear that in everyday life whole-body exposure is not expected. Kift *et al*. (61) reporting on exposure patterns from studies (22, 23) and other UK studies showed that across a range of skin types and ages, the maximum reported skin exposure was for White Caucasian adults in summer at weekends when the median (interquartile range (IQR)) skin area exposed was 17(14–26)%.

Three studies (including four cohorts) measured sun exposure with polysulphone film badges for 1 week per season and sampled for 25OHD in each season. Sun exposure, and the circulating 25OHD, follow a seasonal cycle at UK latitudes, with 25OHD representing the cumulative UV exposure over several weeks before the blood sample is taken. Maximum (summer) and minimum (winter) values are shown above for both sun exposure and 25OHD. Within this constantly changing cycle, winter nadir 25OHD status is to a large extent determined by the late summer peak, which in turn depends on summer sun exposure. Therefore, summer to winter change in 25OHD was equated with summer UV exposure. The results are remarkably consistent, with the two Manchester studies (different years, different cohorts, different ages) falling very close to each other. The two cohorts from Surrey (21) show less apparent response to UV exposure. For one group, the South Asian women, this is explained by skin type and is consistent with other studies showing that skin type 5 requires two to three times more UV than White Caucasian skin types to produce the same vitamin D (49). The postmenopausal Caucasian cohort appears at first glance to be different, but this group has by far the greatest exposure and reaches the highest vitamin D status. It is known that the efficiency of vitamin D synthesis declines with prolonged exposure and that circulating 25OHD plateaus with continuous UV exposure. It is consistent with the evidence that either or both of these effects may be occurring and some of the UV exposure has therefore been ‘wasted’ in terms of vitamin D synthesis. Alternatively, this cohort may have exposed less skin area than other cohorts or may have somewhat decreasing 7DHC capacity in the skin as they are all close to the top of the age range for inclusion. However, these latter two arguments would also apply to the South Asian cohort and if this was the case one would expect a greater difference between the two cohorts.

This analysis is not the same as a direct before–after exposure from artificial sources but is nonetheless representative of practical year-round natural exposures, at least at a location where there is a prolonged vitamin D winter, so that winter can represent ‘before UV’ and summer can represent ‘after UV’. The only solar UV exposure study with a pre-specified dose (2 SED) comes from Brazil (33) and provides a consistent result in terms of response/SDD. While this was a single dose it was in the context of participants at a conference who had presumably had some regular sun exposure prior to the study date and is more representative of seasonal dosing regimes than a UV dose from an artificial source given in winter, isolated from other UV input.

The time in the sun studies are much harder to analyse collectively, being disparate in location, duration, exposure doses, ethnicity, skin type and age. With one exception, they all showed an increase in 25OHD after prescribed UV exposure (more than participants would normally experience). The exception was a study of Thai women (29), comparing UV exposure only with UV exposure and a supplement. Results showed that for the UV exposure group, their monitored exposure time for the study was only 5 min per day more than their normal everyday exposure. Furthermore, the study took place in the rainy season when UV is at its lowest. Thus, it appears that despite the study aims the women were very likely getting less UV exposure than in the rest of the year, and so it is not surprising that their 25OHD status declined.

The clearest response to sun exposure was that of Arab women (30) since the majority of them declared they normally had zero sun exposure. They were asked to expose face and full arms to the sun, in private, for 15 min in the middle of the day, twice a week for 4 weeks. This limited exposure increased 25OHD by 5.4 nmol/L over the study period and shows the advantage of encouraging even short exposures in a vitamin D-deficient cohort.

A group of non-Western immigrants (skin types 4–6) in the Netherlands were followed for 6 months (25OHD in March, June and September, covering the summer half year with sufficient sun for vitamin D synthesis) (31). According to exposure diaries, they had a mean of 2 h exposure to sunlight daily (time of day not specified), both at baseline and after 3 months, exposing hands and face, with just under half exposing forearms. In June, 25OHD had risen by 9.7 nmol/L from a low starting level, but by September, after 3 months of declining solar intensity 7 nmol/L of that increase had been lost. This underscores that highly pigmented individuals can make some vitamin D in their skin with exposure to middle/high latitude sunlight but struggle to reach or maintain a sufficient level for much of the year.

Indian men in Pune were able to increase vitamin D status over 6 months by spending 20 min in the sun in the middle part of the day (27). A similar increase was achieved over 8 weeks by a Korean cohort (32), provided they spent more than 30 min in the sun daily. Compliance in this latter study was poor, and those spending less than 20 min in the sun per day showed little improvement in 25OHD status, especially if the rolling 2-month period was not in the middle of summer.

Finally, two studies had infant, breastfed, participants. In an Indian study (26), the mothers’ vitamin D status was clearly deficient and used as the baseline 25OHD. After 6 months of rather low sun exposure (17 min to 6% skin area daily), infant 25OHD had increased but remained low. By contrast, a study in Beijing (28) exposed the hands and face of infants for about 2 h per day for 2 months in September and October. Their already good 25OHD (70 nmol/L) increased further to 100 nmol/L.

### Dose–response relationship

**Figure 4:**
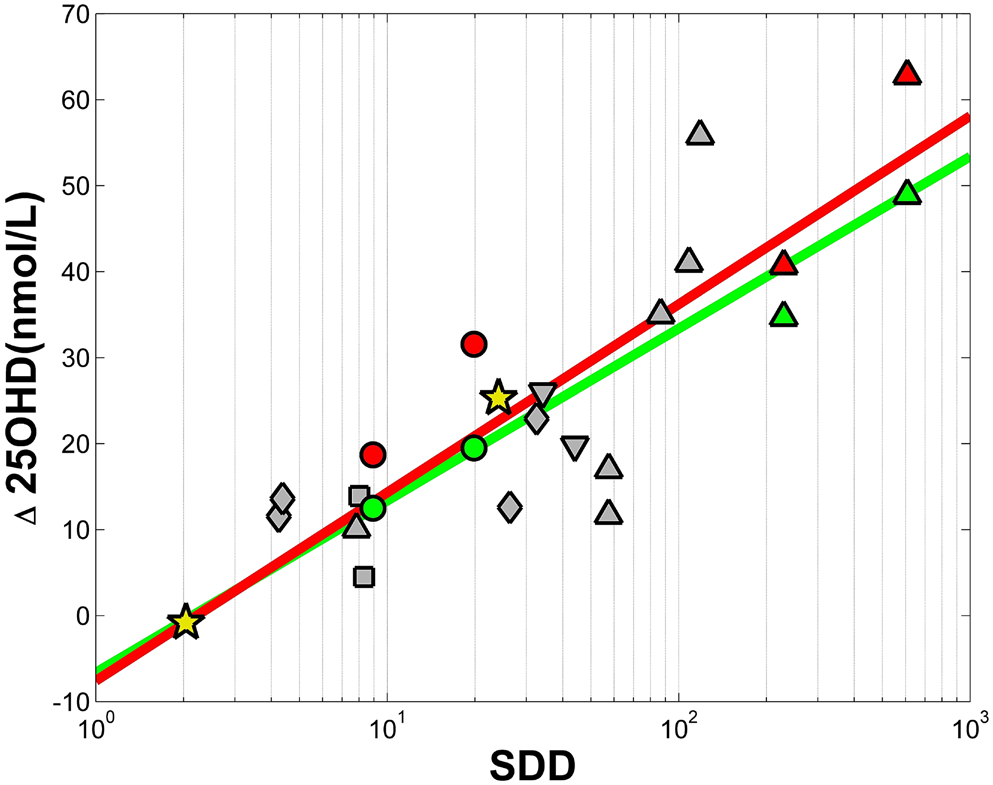
Change in 25 hydroxyvitamin D (Δ25OHD) as a function of standard vitamin D dose (SDD) for sub-erythemal, whole-body exposures to all artificial sources of UV. The red and green points are from study (20): raw data (green points) are used only for the green line dose–response relationship, while seasonally adjusted data (red points) are used only for the red line dose–response relationship. Grey points are all other data, achieved during winter months and using the full duration of UV exposures from the original publications. Symbols indicate the source of UV radiation: upward triangle, TL-01; circle, TL-12; diamond, UV6; square, Arimed B and/or Cleo Natural; downward triangle, Wolff. The yellow stars are estimates from sunlight studies: bottom left from study (34) and centre from studies (61, 68) (see discussion).

Data from all studies with sub-erythemal UV dosing by sources of artificial UV radiation have been combined in an attempt to provide a dose–response relationship. Since skin area exposed is one of the determining factors in response (41, 43), only studies with whole-body exposures are included in Fig. 4. As it is not clear whether all skin synthesises vitamin D equally, care must be taken in extrapolating these results to other skin areas. All but one of the whole-body exposure studies had either all White Caucasian participants, or majority White Caucasian participants. Where mixed skin type participants were included, dose was applied according to skin type. Only one study was focused on skin type 5 and gave three doses of 7 SED (close to MED) on 3 consecutive days. No skin type 6 studies are included in Fig. 4.

The dose–responses derived from Fig. 4 are:


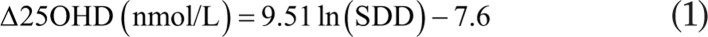


This is the red line in Fig. 4 using the seasonally adjusted data (red points) from (20), with *r*^2^ = 0.66, and


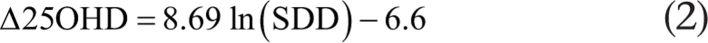


Derived using the raw data from study (20) (green points and green line), with *r*^2^ = 0.52

Note that these results inherently include dietary vitamin D, but not supplements. Where dietary intake was assessed, it was low and consistent throughout the year, for example, studies (21, 22).

## Discussion

The aims of this systematic review were to quantify the impact of UV exposure on vitamin D status and determine a dose–response relationship between UV dose and change in circulating 25OHD. The many factors that influence a dose–response at the personal level have been illustrated by the collected works identified in the review. A dose–response for sub-erythemal, whole-body exposures (~90% skin surface area) to multiple doses of UV has been derived for the first time across a broad range of studies and sources of UV, including SSR.

Consistent messages from the entire collection of studies retained after data extraction show that:

Low dose, sub-erythemal doses are more effective for vitamin D synthesis than doses close to a personal minimum erythema dose. Short, frequent (e.g. daily or several times per week) exposures maximise vitamin D while minimising the risk of skin damage (54, 62).

Increasing the skin area exposed increases the amount of vitamin D synthesised. However, it is not clear whether all skin sites synthesise vitamin D equally, and this – or some other limit – may explain indications that there is not a linear scaling of skin area and change in 25OHD (43). Thus, care must be taken in extrapolating a whole-body dose–response to other exposure regimes.

Constant dosing leads to a plateau in 25OHD, though the level of plateau is dose dependent. To continue to increase vitamin D status, dose must increase. The response to UV dose also depends to some extent on starting levels of 25OHD, though this is most apparent at low vitamin D status (35, 36, 37, 39). A final related statement is that dose–response is greatest at the start of a dosing regime (or for a short duration study vs long duration). Few studies detailed anything more than start and finish 25OHD so these impacts are incorporated into the uncertainty in the dose–response relationship.

There is a large interpersonal variation in response to UV exposure, even accounting for the effects and unknowns above. Caution is required when comparing treatments between small groups of participants.

Solar studies, while more complex and less precisely quantified, show that solar UV exposure is subject to the same general principles. Little and often sun exposure, on maximum skin area that convenience allows, in the middle of the day (at least at temperate latitudes) is the most effective prescription for vitamin D synthesis (34).

We also recognise a number of other potential impacts on the 25OHD response to UV radiation. Studies used to determine the UVR-25OHD dose–response involved volunteers with unprotected skin UVR exposure, and the majority had a normal BMI, with a BMI range representative of the population in the larger sample sizes (20, 47). In real life, a variety of factors including use of sunscreens and obesity could influence the UVR-25OHD relationship (3, 63). A limitation of our work concerns the reliability and comparability of the 25OHD data, in view of inter-laboratory and assay-specific differences in 25OHD measurement (63).

Despite the above variables, sunlight exposure remains a major source of vitamin D, and vitamin D status declines if individuals are deprived of sunlight (64), unless sufficient alternative source of the vitamin is provided. When sunlight is available, determining the exposure parameters to enable suitable vitamin D synthesis is dependent on location, personal characteristics and behaviour. However, with sufficient input data, these can be constrained as illustrated by studies (65, 66), based on earlier human data (47, 49), that indicate ~10 min of sun exposure at lunchtime in UK for white-skinned individuals wearing season-appropriate clothing and ~25 min for skin type 5.

A dose–response (Δ25OHD per UV dose) as derived here provides useful information for the debate on reducing the prevalence of vitamin D deficiency. However, the conditions of the dose–response must be constrained due to the variables of skin photobiology. Additionally, there are no available studies with suitably standardised and quantified doses of solar UV exposure on which to base a dose–response analysis, and so studies using artificial sources of UV have been used. However, the advantage of artificial sources, for the purposes of this assessment, is that they allow a specific, quantified dose of UV to be delivered.

To enable comparison between studies with different lamps, enough detail should be available to allow the VDE dose delivered to be calculated, as performed in this review: the source must be accurately defined within the publication (ideally with spectral characteristics), and the dose measured in a meaningful way with the units and any biological and/or instrument response function clearly stated. Further challenges to direct comparison of different sources exist, especially when doses are large and photochemistry beyond the initial conversion of 7DHC to previtamin D has been possible (Fig. 1). Also, the isomer mixture in skin changes with irradiating spectrum, altering the total amount of previtamin D (and hence finally vitamin D) that a source might enable (10). This cannot be accounted for by expressing the dose in SDD, but the impact is reduced if individual exposures are short and photochemistry beyond previtamin D synthesis is limited. It is clear from Fig. 4 that no one source provides for systematically high or low 25OHD response compared with the others. This provides a level of confidence both in our decision to limit the dose–response to short exposures and in the action spectrum for previtamin D synthesis that we have used.

The dose–response (red line) in Fig. 4 provides a link between UV dose and change in 25OHD represented by the equation:


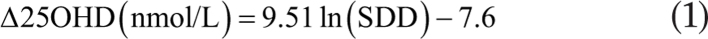


This is in reasonable agreement with study (20), though note 2 of the data points (red in Fig. 4) used in equation (1) come from this publication so the results are not entirely independent. Their dose–response, based on data from two lamps at two time points, and with seasonally adjusted data (also used in deriving equation (1)) was:


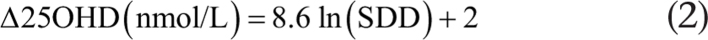


The ‘seasonally adjusted data’ was a model adjustment to account for sun exposure of participants during the rolling recruitment in the original study and was provided in the publication (20). The authors of (20) also added an ‘origin’ point to their regression at close to (0,0) but avoiding errors with logarithms (R McKenzie, personal communication). We have not taken this route in Fig. 4, on the basis that with no UV exposure vitamin D will decline, rather than stay constant. Adding an ‘origin’ point at (1,0) to Fig. 4 to match study (20) would result in equation (1) becoming:


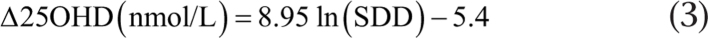


which is then in good agreement with study (20) despite the addition of many other studies to the regression.

Using the raw data from study (20) in our calculation (Fig. 4 green points and green line, no origin point) reduces both the slope and intercept of our regression line as shown in equation (2).

The consistency in the above results can be summarised in a single dose–response of the form:


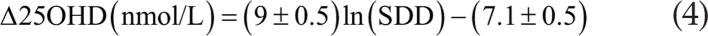


This applies strictly to equations (1) and (2), and in all but intercept to equations (3) and (4) that had an origin point included. The intercept is hard to interpret but as noted above would not be expected to go through the origin.

The authors of study (67) also attempt a dose–response relationship, from otherwise unpublished data using a UV6 lamp and a series of doses accumulating to 10 SED, so at the lower range of the studies included here. They find a linear dose–response relation at these doses, as indeed could current authors if only low accumulated dose studies are included. However, the (assumed mean) change in 25OHD in (67) is much greater than observed in the studies identified for this review, at ~50 nmol/L after 10 SED (5 × 2 SED) of full-body exposure, and does not easily fit our dose–response curve. The closest comparative study in our review was study (44), also providing five doses from a UV6 lamp over about 2 weeks but with each dose of 0.85 SDD rather than 3.1 SDD (2 SED): study (44) is identified as the lowest cumulative dose in Fig. 4. It is worth noting that the lower personal Δ25OHD in (67) (individual points identified) would fit neatly into our dose–response, and several authors have identified the large interpersonal differences in response to UV radiation (24) so it is possible that the small number of participants (10) represented in (67) has resulted in this different dose–response relationship.

While we have been able to construct a dose–response relationship between whole-body UV dose from a range of phototherapy sources and Δ25OHD, it still remains to translate this into an everyday setting. For the great majority of people, the sun is their only source of UV radiation, and in daily life is accessed intermittently and with limited skin area exposed. The sun exposure studies (Table 5) show that sun exposure on limited, although not fully quantified, skin surface area can and does increase vitamin D status. Where it fails to do so, the exposure was less than that which induced the starting 25OHD (29), or exposure was minimal (25) and starting 25OHD taken from mother not infant. The generally unquantified skin area exposed, and lack of long-term monitoring of exposure, precludes calculations equivalent to those for artificial sources. Nonetheless, whole-body exposures from SSR (Table 4) are incorporated into Fig. 4, giving some confidence that whole-body sunlight exposures would also fit the dose–response relationship, if they could be precisely quantified.

The impact of reducing the skin area exposed from whole body to the more practical everyday range of 10–35% (representing hands and face only or attire equivalent to modest shorts and T-shirt) is difficult to assess. Simple scaling by skin area exposed may not be appropriate; indeed, there is evidence that such scaling is not appropriate (41, 43, 67), though note that all these studies used small groups of different people exposing different skin areas, which could distort the results. A longitudinal study would be more informative. There is also an argument that skin synthesis of vitamin D may be different between regularly exposed skin areas and those that are habitually covered because of changes in pigment and epidermal thickness for regularly exposed skin. This too needs further clarification.

Despite the above caveats, we can make a crude attempt to confirm whether or not the above dose–response relationship might also apply to sunlight using the data from studies (61, 68) that both used data from the same original study. Mean values from the participant cohort of 109 White Caucasian adults are used – just as average (mean or median) values were used in constructing Fig. 4. Total summer sun exposure increased 25OHD by a mean of 25.3 nmol/L. Exposure in the monitored weeks in spring (April) and summer (July) was on average 4 SED/week, reducing to 1 SED by October and 0.1 SED in January (65). We estimate total exposure across the summer to be 96 SED (181 SDD for Manchester summer sun). Median skin area exposed was 11% in spring and 14% in summer (IQR 8–17% in both seasons) (59). We take median skin area exposed across the spring and summer to be 12% and scale exposure from whole body (actually ~90% skin area) to 12% in a linear fashion, resulting in an effective 24.1 SDD. The point (24.1, 25.3) is shown by the middle star in Fig. 4.

A further sunlight data point can be added from study (34). This was a study of 512 New Zealanders of four different ethnicities covering skin types 1–4. A quarter of participants were older than our age cut-off of 65 years, but they cannot be separated from the other participants in the results and so are accepted here for inclusion. We have taken the median sun exposure recorded for a 4-week period, scaled up to full-body exposure by the original authors, and converted from SED to SDD using an estimated scale factor of 1.5 (see Table 1, all exposures in the autumn, winter, spring period). This is plotted in Fig. 4 against the median change in 25OHD over the same 4-week period. The point (2.04, −0.8) is shown by the lower left star in Fig. 4 and illustrates the approximate threshold at which long-term, low-dose UV ceases to maintain vitamin D status. An alternative calculation by the original authors, using the 512 participants stratified by sun exposure, estimated the threshold to be 0.5 SED/week (full-body equivalent exposure).

These two sunlight data points are not out of place in Fig. 4, and even changing the estimated SDD by up to 50% in either direction would leave the solar data fitting the dose–response as well as the underlying lamp studies on which it is based. This gives some confidence that the dose–response relationship derived from controlled studies with artificial sources of UV radiation is not unrepresentative of the same response in sunlight, provided that both units of exposure and skin area exposed can be suitably specified.

The limitation that the dose–response is based on predominantly White Caucasian skin types persists. Farrar *et al.* (49) showed that skin type 5 people need about 2.5 times as much simulated sun exposure as white-skinned people, delivered as small doses three times weekly, to enable equivalent vitamin D synthesis, which implies that equation (1) may not be appropriate to non-white skin types. More data on only skin types 5 and 6 are required to confirm this and allow targeted sun exposure guidance for different sub-groups of the population, as recommended by study (63).

## Conclusion

There are relatively few intervention studies that explore vitamin D synthesis following UV exposure while fully quantifying the UV dose and unprotected skin area exposed. There is no such study of solar exposure over a prolonged period, which may reflect the difficulties of constraining the variables. There is also limited data on non-white skin types from studies of either solar or artificial UV sources; thus, the quantitative results here are applicable to white skin. There is no reason to believe that the more qualitative findings do not apply to all skin types.

Findings include that small UV doses on a regular basis are more efficient for vitamin D synthesis than larger sub-erythemal doses; that darker skin needs a larger UV dose to give the same absolute change in 25OHD; that even accounting for different skin types there are large interpersonal differences in response to UV exposure; and increasing UV doses over a longer period gives greater overall efficacy of 25OHD production.

A dose–response relationship, based on whole-body exposure to a range of artificial sources of UV has been determined, following definition of the dose delivered in SDD to account for the spectra of the different sources. The dosing regimes and duration of the underlying studies varied considerably, but all used sub-erythemal doses of UV. The dose–response may also apply to solar exposure, although this is subject to a number of caveats. Nonetheless, it can provide a guide to exposure regimes that enhance vitamin D status while limiting risks from sun exposure.

This work synthesises and extends knowledge of UV impact on vitamin D status and is informative for updates on guidance for human nutrition in vitamin D.

## Supplementary Material

Supplementary Table 1 Embase (including Medline) Search Terms (19 October 2020) and Cochrane Central Search Terms (19 October 2020)

Supplementary Table 2 Inclusion/exclusion criteria

## Declaration of interest

The authors declare that there is no conflict of interest that could be perceived as prejudicing the impartiality of this review.

## Funding

This work was supported by funding from FAO and WHO. R A is supported by a PhD studentship from King Abdulaziz University, Jeddah, Saudi Arabia. L E R acknowledges support of the NIHR Manchester Biomedical Research Centre.

## References

[1] Lucas RM Yazar S Young AR Norval M de Gruijl FR Takizawa Y Rhodes LE Sinclair CA Neale RE. Human health in relation to exposure to solar ultraviolet radiation under changing stratospheric ozone and climate. Photochemical and Photobiological Sciences 2019 18 641–680. (10.1039/c8pp90060d) 30810559

[2] Itkonen ST Andersen R Björk AK Brugård Konde Å Eneroth H Erkkola M Holvik K Madar AA Meyer HE Tetens I Vitamin D status and current policies to achieve adequate vitamin D intake in the Nordic countries. Scandinavian Journal of Public Health 2020 1403494819896878. (10.1177/1403494819896878) 31916497

[3] SACN. The Scientific Advisory Committee on Nutrition (SACN) Recommendations on Vitamin D. Public Health of England, 2016. (available at: https://assets.publishing.service.gov.uk/government/uploads/system/uploads/attachment_data/file/537616/SACN_Vitamin_D_and_Health_report.pdf>)

[4] Institute of Medicine (US). Dietary Reference Intakes for Calcium and Vitamin D Washington, DC, USA: National Academies Press (US), 2011. (10.17226/13050) 21796828

[5] EFSA. Dietary reference values for vitamin D. EFSA Journal 2016 14 e04547. 10.2903/j.efsa.2017.4780 PMC7010012 32625486

[6] Cashman KD Dowling KG Skrabakova Z Gonzalez-Gross M Valtuena J De Henauw S Moreno L Damsgaard CT Michaelsen KF Molgaard C Vitamin D deficiency in Europe: pandemic? American Journal of Clinical Nutrition 2016 103 1033–1044. (10.3945/ajcn.115.120873) PMC5527850 26864360

[7] Hilger J Friedel A Herr R Rausch T Roos F Wahl DA Pierroz DD Weber P Hoffmann K. A systematic review of vitamin D status in populations worldwide. British Journal of Nutrition 2014 111 23–45. (10.1017/S0007114513001840) 23930771

[8] Webb AR Kline L Holick MF. Influence of season and latitude on the cutaneous synthesis of vitamin D3: exposure to winter sunlight in Boston and Edmonton will not promote vitamin D3 synthesis in human skin. Journal of Clinical Endocrinology and Metabolism 1988 67 373–378. (10.1210/jcem-67-2-373) 2839537

[9] Webb AR Decosta BR Holick MF. Sunlight regulates the cutaneous production of vitamin D3 by causing its photodegradation. Journal of Clinical Endocrinology and Metabolism 1989 68 882–887. (10.1210/jcem-68-5-882) 2541158

[10] MacLaughlin JA Anderson RR Holick MF. Spectral character of sunlight modulates photosynthesis of previtamin *D*-sub-3 and its photoisomer in human skin. Science 1982 216 1001–1003. 6281884 10.1126/science.6281884

[11] Bouillon R Schuit F Antonio L Rastinejad F. Vitamin D binding protein: a historic overview. Frontiers in Endocrinology 2019 10 910–910. (10.3389/fendo.2019.00910) 31998239 PMC6965021

[12] MacLaughlin JaH Holick MF. Aging decreases the capacity of human skin to produce vitamin D3. Journal of Clinical Investigation 1985 76 1536–1538. (10.1172/JCI112134) PMC424123 2997282

[13] Webb AR Campbell GA Steven MS Hosking DJ. Correction of vitamin D deficiency in elderly long-stay patients by sunlight exposure. Bone 1990 12 296–297.

[14] Hirani V Primatesta P. Vitamin D concentrations among people aged 65 years and over living in private households and institutions in England: population survey. Age and Ageing 2005 34 485–491. (10.1093/ageing/afi153) 16043444

[15] Borecka O Farrar MD Osman JE Rhodes LE Webb AR. Older adults who spend more time outdoors in summer and have higher dietary vitamin D Than younger adults can present at least as high vitamin D status: a pilot study. International Journal of Environmental Research and Public Health 2021 18 3364. (10.3390/ijerph18073364) PMC8037349 33805086

[16] CIE. Action Spectrum for the Production of Previtamin D3 in Human Skin, CIE 174:2006. 2006.

[17] McKenzie RL Liley JB Björn LO. UV radiation: balancing risks and benefits. Photochemistry and Photobiology 2009 85 88–98. (10.1111/j.1751-1097.2008.00400.x) 18657052

[18] Norval M Björn LO de Gruijl FR. Is the action spectrum for the UV-induced production of previtamin d3 in human skin correct? Photochemical and Photobiological Sciences 2010 9 11–17. (10.1039/b9pp00012g) 20062839

[19] van Dijk A den Outer P van Kranen H Slaper H. The action spectrum for vitamin D3: initial skin reaction and prolonged exposure. Photochemical and Photobiological Sciences 2016 15 896–909. (10.1039/c6pp00034g) 27286277

[20] McKenzie R Scragg R Liley B Johnston P Wishart J Stewart A Prematunga R. Serum 25-hydroxyvitamin-D responses to multiple UV exposures from solaria: inferences for exposure to sunlight. Photochemical and Photobiological Sciences 2012 11 1174–1185. (10.1039/c2pp05403e) 22411223

[21] Darling AL Hart KH Macdonald HM Horton K Kang’ombe AR Berry JL Lanham-New SA. Vitamin D deficiency in UK South Asian Women of childbearing age: a comparative longitudinal investigation with UK Caucasian women. Osteoporosis International 2013 24 477–488. (10.1007/s00198-012-1973-2) 22525977

[22] Rhodes LE Webb AR Berry JL Felton SJ Marjanovic EJ Wilkinson JD Vail A Kift R. Sunlight exposure behaviour and vitamin D status in photosensitive patients: longitudinal comparative study with healthy individuals at U.K. latitude. British Journal of Dermatology 2014 171 1478–1486. (10.1111/bjd.13325) 25110159

[23] Farrar MD Mughal MZ Adams JE Wilkinson J Berry JL Edwards L Kift R Marjanovic E Vail A Webb AR Sun exposure behavior, seasonal vitamin D deficiency, and relationship to bone health in adolescents. Journal of Clinical Endocrinology and Metabolism 2016 101 3105–3113. (10.1210/jc.2016-1559) 27228370

[24] Datta P Philipsen PA Olsen P Petersen B Johansen P Morling N Wulf HC. Major inter-personal variation in the increase and maximal level of 25-hydroxy vitamin D induced by UVB. Photochemical and Photobiological Sciences 2016 15 536–545. (10.1039/c5pp00462d) 27001558

[25] Rueter K Jones AP Siafarikas A Lim EM Bear N Noakes PS Prescott SL Palmer DJ. Direct infant UV light exposure is associated with eczema and immune development. Journal of Allergy and Clinical Immunology 2019 143 1012.e2–1020.e2. (10.1016/j.jaci.2018.08.037) 30366577

[26] Meena P Dabas A Shah D Malhotra RK Madhu SV Gupta P. Sunlight exposure and vitamin D status in breastfed infants. Indian Pediatrics 2017 54 105–111. (10.1007/s13312-017-1010-9) 28031546

[27] Patwardhan VG Mughal ZM Padidela R Chiplonkar SA Khadilkar VV Khadilkar AV. Randomized control trial assessing impact of increased sunlight exposure versus vitamin D supplementation on lipid profile in indian vitamin D deficient men. Indian Journal of Endocrinology and Metabolism 2017 21 393–398. (10.4103/ijem.IJEM_9_17) 28553593 PMC5434721

[28] Ho ML Yen HC Tsang RC Specker BL Chen XC Nichols BL. Randomized study of sunshine exposure and serum 25-OHD in breast-fed infants in Beijing, China. Journal of Pediatrics 1985 107 928–931. (10.1016/s0022-3476(8580192-x) 4067751

[29] Watcharanon W Kaewrudee S Soontrapa S Somboonporn W Srisaenpang P Panpanit L Pongchaiyakul C. Effects of sunlight exposure and vitamin D supplementation on vitamin D levels in postmenopausal women in rural Thailand: a randomized controlled trial. Complementary Therapies in Medicine 2018 40 243–247. (10.1016/j.ctim.2018.06.004) 30219459

[30] Dawodu A Kochiyil J Altaye M. Pilot study of sunlight exposure and vitamin D status in Arab women of childbearing age. Eastern Mediterranean Journal 2011 17 570–574. 21972479

[31] Wicherts IS Boeke AJ van der Meer IM van Schoor NM Knol DL Lips P. Sunlight exposure or vitamin D supplementation for vitamin D-deficient non-western immigrants: a randomized clinical trial. Osteoporosis International 2011 22 873–882. (10.1007/s00198-010-1343-x) 20683712 PMC3034877

[32] Joh HK Hwang SS Cho B Lim CS Jung SE. Effect of sun exposure versus oral vitamin D supplementation on serum 25-hydroxyvitamin D concentrations in young adults: a randomized clinical trial. Clinical Nutrition 2020 39 727–736. (10.1016/j.clnu.2019.03.021) 30987813

[33] Pereira LA Luz FB Carneiro CMMO Xavier ALR Kanaan S Miot HA. Evaluation of vitamin D plasma levels after mild exposure to the sun with photoprotection. Anais Brasileiros de Dermatologia 2019 94 56–61. (10.1590/abd1806-4841.20198070) PMC6360984 30726465

[34] Scragg RKR Stewart AW McKenzie RL Reeder AI Liley JB Allen NW. Sun exposure and 25-hydroxyvitamin D3 levels in a community sample: quantifying the association with electronic dosimeters. Journal of Exposure Science and Environmental Epidemiology 2017 22 471–477. 10.1038/jes.2016.51 27599885

[35] Karppinen T Ala-Houhala M Ylianttila L Kautiainen H Viljakainen H Reunala T Snellman E. Narrowband ultraviolet B exposures maintain vitamin D levels during winter: a randomized controlled trial. Acta Dermato-Venereologica 2016 96 490–493. (10.2340/00015555-2269) 26524984

[36] Bogh MK Gullstrand J Svensson A Ljunggren B Dorkhan M. Narrowband ultraviolet B three times per week is more effective in treating vitamin D deficiency than 1600 IU oral vitamin D(3) per day: a randomized clinical trial. British Journal of Dermatology 2012 167 625–630. (10.1111/j.1365-2133.2012.11069.x) 22632734

[37] Bosman ES Albert AY Lui H Dutz JP Vallance BA. Skin exposure to narrow band ultraviolet (UVB) light modulates the human intestinal microbiome. Frontiers in Microbiology 2019 10 2410. (10.3389/fmicb.2019.02410) PMC6821880 31708890

[38] Ponda MP Liang Y Kim J Hutt R Dowd K Gilleaudeau P Sullivan-Whalen MM Rodrick T Kim DJ Barash I A randomized clinical trial in vitamin D-deficient adults comparing replenishment with oral vitamin D3 with narrow-band UV type B light: effects on cholesterol and the transcriptional profiles of skin and blood. American Journal of Clinical Nutrition 2017 105 1230–1238. (10.3945/ajcn.116.150367) PMC5402037 28228421

[39] Ala-Houhala MJ Karppinen T Vahavihu K Kautiainen H Dombrowski Y Snellman E Schauber J Reunala T. Narrow-band ultraviolet B treatment boosts serum 25-hydroxyvitamin D in patients with psoriasis on oral vitamin D supplementation. Acta Dermato-Venereologica 2014 94 146–151. (10.2340/00015555-1685) 23995795

[40] Ala-Houhala MJ Vahavihu K Hasan T Kautiainen H Ylianttila L Viljakainen HT Snellman E Reunala T. Comparison of narrowband ultraviolet B exposure and oral vitamin D substitution on serum 25-hydroxyvitamin D concentration. British Journal of Dermatology 2012 167 160–164. (10.1111/j.1365-2133.2012.10990.x) 22512509

[41] Bogh MKB Schmedes AV Philipsen PA Thieden E Wulf HC. Vitamin D production depends on ultraviolet-B dose but not on dose rate: a randomized controlled trial. Experimental Dermatology 2011 20 14–18. (10.1111/j.1600-0625.2010.01201.x) 21158934

[42] Osmancevic A Demeke T Gillstedt M Angesjo E Sinclair H Abd El-Gawad G Landin-Wilhelmsen K. Vitamin D treatment in Somali women living in Sweden – two randomized, placebo-controlled studies. Clinical Endocrinology 2016 85 535–543. (10.1111/cen.13097) 27155232

[43] Bogh MK Schmedes AV Philipsen PA Thieden E Wulf HC. Interdependence between body surface area and ultraviolet B dose in vitamin D production: a randomized controlled trial. British Journal of Dermatology 2011 164 163–169. (10.1111/j.1365-2133.2010.10082.x) 21039402

[44] Sallander E Wester U Bengtsson E Wiegleb Edstrom D. Vitamin D levels after UVB radiation: effects by UVA additions in a randomized controlled trial. Photodermatology, Photoimmunology and Photomedicine 2013 29 323–329. (10.1111/phpp.12076) 24118541

[45] Yesudian PD Berry JL Wiles S Hoyle S Young DB Haylett AK Rhodes LE Davies P. The effect of ultraviolet *B*-induced vitamin D levels on host resistance to *Mycobacterium tuberculosis*: a pilot study in immigrant Asian adults living in the United Kingdom. Photodermatology, Photoimmunology and Photomedicine 2008 24 97–98. (10.1111/j.1600-0781.2008.00339.x) 18353091

[46] Bogh MK Schmedes AV Philipsen PA Thieden E Wulf HC. A small suberythemal ultraviolet B dose every second week is sufficient to maintain summer vitamin D levels: a randomized controlled trial. British Journal of Dermatology 2012 166 430–433. (10.1111/j.1365-2133.2011.10697.x) 22013924

[47] Rhodes LE Webb AR Fraser HI Kift R Durkin MT Allan D O’Brien SJ Vail A Berry JL. Recommended summer sunlight exposure levels can produce sufficient (≥20 ng ml^-1^) but not the proposed optimal (≥32 ng ml^-1^) 25(OH)D levels at UK latitudes. Journal of Investigative Dermatology 2010 130 1411–1418. (10.1038/jid.2009.417) 20072137

[48] Langdahl JH Schierbeck LL Bang UC Jensen JE. Changes in serum 25-hydroxyvitamin D and cholecalciferol after one whole-body exposure in a commercial tanning bed: a randomized study. Endocrine 2012 42 430–435. (10.1007/s12020-012-9641-z) 22391940

[49] Farrar MD Webb AR Kift R Durkin MT Allan D Herbert A Berry JL Rhodes LE. Efficacy of a dose range of simulated sunlight exposures in raising vitamin D status in South Asian adults: implications for targeted guidance on sun exposure. American Journal of Clinical Nutrition 2013 97 1210–1216. (10.3945/ajcn.112.052639) 23615828

[50] Biersack MG Hajdukiewicz M Uebelhack R Franke L Piazena H Klaus P Hohne-Zimmer V Braun T Buttgereit F Burmester GR Sustained increase of 25-hydroxyvitamin D levels in healthy young women during wintertime after three suberythemal UV irradiations-the MUVY pilot study. PLoS ONE 2016 11 e0159040. (10.1371/journal.pone.0159040) PMC4951026 27434043

[51] Lagunova Z Porojnicu AC Aksnes L Holick MF Iani V Bruland OS Moan J. Effect of vitamin D supplementation and ultraviolet B exposure on serum 25-hydroxyvitamin D concentrations in healthy volunteers: a randomized, crossover clinical trial. British Journal of Dermatology 2013 169 434–440. (10.1111/bjd.12349) 23551243

[52] Carbone LD Rosenberg EW Tolley EA Holick MF Hughes TA Watsky MA Barrow KD Chen TC Wilkin NK Bhattacharya SK 25-Hydroxyvitamin *D*, cholesterol, and ultraviolet irradiation. Metabolism: Clinical and Experimental 2008 57 741–748. (10.1016/j.metabol.2008.01.011) 18502255

[53] Porojnicu AC Bruland OS Aksnes L Grant WB Moan J. Sun beds and cod liver oil as vitamin D sources. Journal of Photochemistry and Photobiology: B, Biology 2008 91 125–131. (10.1016/j.jphotobiol.2008.02.007) 18417354

[54] Felton SJ Cooke MS Kift R Berry JL Webb AR Lam PM de Gruijl FR Vail A Rhodes LE. Concurrent beneficial (vitamin D production) and hazardous (cutaneous DNA damage) impact of repeated low-level summer sunlight exposures. British Journal of Dermatology 2016 175 1320–1328. (10.1111/bjd.14863) PMC5215649 27411377

[55] CIE. Erythema reference action spectrum and standard erythema dose. CIE 1998 7 E1998.

[56] Vieth R Vitamin D and cancer mini-symposium: the risk of additional vitamin D. Annals of Epidemiology 2009 19 441–445. (10.1016/j.annepidem.2009.01.009) 19364661

[57] McKenzie R Blumthaler M Diaz S Fioletov V Herman J Seckmeyer G Smedley A Webb A Rationalizing nomenclature for UV doses and effects on humans CIE 209:2014/WMO/GAW Report No. 211 ISBN 978-3-902842-35-0, 2014.

[58] MATLAB and Statistics Toolbox Release MATLAB R2020a. Natick, MA, USA: T.M., Inc. (available at: https://uk.mathworks.com/products/new_products/release2020a.html)

[59] Weber B Marculescu R Radakovic S Tanew A. Serum levels of folate, 25-hydroxyvitamin D3 and cobalamin during UVB phototherapy: findings in a large prospective trial. Journal of the European Academy of Dermatology and Venereology 2020 34 385–391. (10.1111/jdv.15941) 31494977 PMC7027503

[60] Staberg B Christiansen C Rossing N. Serum vitamin D metabolites in normal subjects after phototherapy. Scandinavian Journal of Clinical and Laboratory Investigation 1984 44 53–56. (10.3109/00365518409083787) 6608135

[61] Kift R Rhodes LE Farrar MD Webb AR. Is sunlight exposure enough to avoid wintertime vitamin D deficiency in united kingdom population groups? International Journal of Environmental Research and Public Health 2018 15 1624. (10.3390/ijerph15081624) PMC6121420 30071636

[62] Shih BB Farrar MD Cooke MS Osman J Langton AK Kift R Webb AR Berry JL Watson REB Vail A Fractional sunburn threshold UVR doses generate equivalent vitamin D and DNA damage in skin Types I–VI but with epidermal DNA damage gradient correlated to skin darkness. Journal of Investigative Dermatology 2018 138 2244–2252. (10.1016/j.jid.2018.04.015) PMC6158343 29730334

[63] AGNIR. UV Radiation Vitamin D Health: Report of the Independent Advisory Group on Non-Ionising Radiation . London, UK: Public Health England, 2017. (available at: https://assets.publishing.service.gov.uk/government/uploads/system/uploads/attachment_data/file/620184/UV_Radiation_Vitamin_D_Health.pdf)

[64] Iuliano-Burns S Wang XF Ayton J Jones G Seeman E. Skeletal and hormonal responses to sunlight deprivation in Antarctic expeditioners. Osteoporosis International 2009 20 1523–1528. (10.1007/s00198-008-0830-9) 19151911

[65] Webb AR Kazantzidis A Kift RC Farrar MD Wilkinson J Rhodes LE. Colour counts: sunlight and skin type as drivers of vitamin D deficiency at UK latitudes. Nutrients 2018 10 457. (10.3390/nu10040457) PMC5946242 29642423

[66] Webb AR Kazantzidis A Kift RC Farrar MD Wilkinson J Rhodes LE. Meeting vitamin D requirements in white caucasians at UK latitudes: providing a choice. Nutrients 2018 10 497. (10.3390/nu10040497) PMC5946282 29673142

[67] Neville JJ Palmieri T Young AR. Physical determinants of vitamin D photosynthesis: a review. JBMR Plus 2021 5 e10460. (10.1002/jbm4.10460) PMC7839826 33553995

[68] Webb AR Kift R Durkin MT O’Brien SJ Vail A Berry JL Rhodes LE. The role of sunlight exposure in determining the vitamin D status of the U.K. white adult population. British Journal of Dermatology 2010 163 1050–1055. (10.1111/j.1365-2133.2010.09975.x) 20716215

[69] Scragg R Wishart J Stewart A Ofanoa M Kerse N Dyall L Lawes CM. No effect of ultraviolet radiation on blood pressure and other cardiovascular risk factors. Journal of Hypertension 2011 29 1749–1756. (10.1097/HJH.0b013e328349666d) 21720260

