# Supplementary material for: 100 YEARS OF VITAMIN D: Dose–response for change in 25-hydroxyvitamin D after UV exposure: outcome of a systematic review
Source: Endocr Connect. 2021 Sep 13;10(10):R248–66. doi: 10.1530/EC-21-0308 (PMC8558903; doi:10.1530/EC-21-0308)
Supplement: Supplementary Table 1 Embase (including Medline) Search Terms (19 October 2020) and Cochrane Central Search Terms (19 October 2020) [file supplementary_table_1.pdf]

Supplementary Table 1 Embase (including Medline) Search Terms (19 October 2020) and Cochrane Central Search Terms (19 October 2020)

#	Search terms	Result
1	25OHD$ OR '25-OH' OR '25-hydroxy vitamin' OR '25-hydroxyvitamin' OR '25-dihydroxyvitamin' OR calcifediol OR '25-hydroxycholecalciferol' OR '25-hydroxy cholecalciferol' OR (('vitamin D$' OR cholecalciferol$ OR ergocalciferol$ OR calciferol OR calcidol) NEAR/2 level$) OR ('vitamin D$' NEAR/2 (metabolism OR status))	71,568
2	'sunlight'/exp OR 'ultraviolet radiation'/exp OR 'solar radiation'/exp OR sunlight OR 'sun light' OR sunshine OR 'sun shine' OR solar OR ultraviolet OR UVR OR UV-R OR UVB OR UV-B OR 'artificial light' OR (exposure NEAR/3 sun)	335,713
3	1 AND 2	7,311
4	'clinical trial'/exp OR 'intervention study'/exp OR 'experimental study'/exp OR 'open study'/exp OR 'major clinical study'/exp OR 'pilot study'/exp OR 'feasibility study'/exp	5,238,573
5	3 AND 4	2,147
6	('animal'/exp OR 'nonhuman'/exp OR 'animal cell'/exp OR 'animal cell culture'/exp OR ‘animal experiment’/exp OR ‘animal tissue’/exp OR ‘animal model’/exp) NOT (('animal'/exp OR 'nonhuman'/exp OR 'animal cell'/exp OR 'animal cell culture'/exp OR ‘animal experiment’/exp OR 'animal tissue'/exp OR 'animal model'/exp) AND 'human'/exp)	7,191,854
8	5 NOT 6	2,123
9	8 NOT 'conference abstract'/it	1,658

Cochrane CENTRAL (19 October 2020)

#	Search terms	Result
1	25OHD$ OR “25$OH” OR “25$hydroxy vitamin D$” OR “25$hydroxyvitamin D$” OR calcifediol OR “25$hydroxycholecalciferol” OR “25$hydroxy cholecalciferol” OR ((“vitamin D$” OR cholecalciferol$ OR ergocalciferol$ OR calciferol OR calcidol) NEAR/2 level$) OR (“vitamin D$” NEAR/2 (metabolism OR status))	6,429
2	sunlight [MeSH] OR ultraviolet radiation [MeSH] OR sunlight OR “sun light” OR sunshine OR “sun shine” OR solar OR ultraviolet OR UVR OR UV$R OR UVB OR UV$B OR “artificial light” OR (exposure NEAR/3 sun)	5474
3	1 AND 2	606*

*Of these 3 are Cochrane reviews and 1 is editorial = 602 trials.

Supplementary Table 2 Inclusion/exclusion criteria

	Included	Excluded
**Population of interest**<br>Apparently healthy children and adults	• Healthy children of any age<br>• Healthy adults up to 65 years of age	• Those with illness that might impact vitamin D or calcium status or metabolism<br>• Pregnancy or lactation
**Outcomes**<br>Measures of vitamin D status	• Serum or plasma 25(OH)D levels	• Studies not reporting outcome of interest<br>• Studies not reporting baseline and endpoint concentrations
**Interventions**<br>Exposure to sunlight or artificial UVB	• For studies with natural sunlight exposure, quantification of personal exposure (personal dosimeter and/or exposure diary and ambient measurements for sunlight)<br>• Defined dose of artificial UV<br>• For studies using artificial UVB, the source must be clearly specified, with spectral information in the paper or available because a standard lamp type.	• Studies providing high-dose UVB for therapeutic purposes (i.e. dose greater than MED (minimum erythema dose)<br>• Studies not quantifying natural sunlight exposure<br>• Studies not reporting source of UVB or sufficient detail regarding the source of UVB
**Study designs**	• Intervention studies; including randomized and non-randomized controlled trials, non-controlled intervention studies (i.e. before and after studies)	• Observational studies including cohort, case-control, cross-sectional, ecological studies<br>• Animal studies<br>• Reviews (but use for snowball referencing).
**Study details**	• Studies of any duration<br>• Minimum sample size of N>2	• Individual/ case-control studies<br>• Studies with participants taking vitamin D supplements
**Publication type**	• Peer-reviewed journal publications	• Conference posters and abstracts<br>• Articles for which full-text unobtainable
**Other**<br>No restrictions on dates, settings, languages (WHO will translate if needed)	• All dates, settings, languages	
