# Supplementary material for: 100 YEARS OF VITAMIN D: Dose–response for change in 25-hydroxyvitamin D after UV exposure: outcome of a systematic review
Source: Endocr Connect. 2021 Sep 13;10(10):R248–66. doi: 10.1530/EC-21-0308 (PMC8558903; doi:10.1530/EC-21-0308)
Supplement: Supplementary Table 2 Inclusion/exclusion criteria [file supplementary_table_2.pdf]

Supplementary Table 2 Inclusion/exclusion criteria

	Included	Excluded
**Population of interest**<br><br>Apparently healthy children and adults	• Healthy children of any age<br>• Healthy adults up to 65 years of age	• Those with illness that might impact vitamin D or calcium status or metabolism<br>• Pregnancy or lactation
**Outcomes**<br><br>Measures of vitamin D status	• Serum or plasma 25(OH)D levels	• Studies not reporting outcome of interest<br>• Studies not reporting baseline and endpoint concentrations
**Interventions**<br><br>Exposure to sunlight or artificial UVB	• For studies with natural sunlight exposure, quantification of personal exposure (personal dosimeter and/or exposure diary and ambient measurements for sunlight)<br>• Defined dose of artificial UV<br>• For studies using artificial UVB, the source must be clearly specified, with spectral information in the paper or available because a standard lamp type.	• Studies providing high-dose UVB for therapeutic purposes (i.e. dose greater than MED (minimum erythema dose)<br>• Studies not quantifying natural sunlight exposure<br>• Studies not reporting source of UVB or sufficient detail regarding the source of UVB
**Study designs**	• Intervention studies; including randomized and non-randomized controlled trials, non-controlled intervention studies (i.e. before and after studies)	• Observational studies including cohort, case-control, cross-sectional, ecological studies<br>• Animal studies<br>• Reviews (but use for snowball referencing).
**Study details**	• Studies of any duration<br>• Minimum sample size of N>2	• Individual/ case-control studies<br>• Studies with participants taking vitamin D supplements
**Publication type**	• Peer-reviewed journal publications	• Conference posters and abstracts<br>• Articles for which full-text unobtainable
**Other**<br><br>No restrictions on dates, settings, languages (WHO will translate if needed)	• All dates, settings, languages	
